# Quantum Models à la Gabor for the Space-Time Metric

**DOI:** 10.3390/e24060835

**Published:** 2022-06-16

**Authors:** Gilles Cohen-Tannoudji, Jean-Pierre Gazeau, Célestin Habonimana, Juma Shabani

**Affiliations:** 1 Laboratoire de Recherche sur les Sciences de la Matière, LARSIM CEA, Université Paris-Saclay, F-91190 Saint-Aubin, France; gilles.cohen-tannoudji@cea.fr; 2 CNRS, Astroparticule et Cosmologie, Université Paris Cité, F-75013 Paris, France; 3 Ecole Normale Supérieure, Université du Burundi, Bujumbura 1550, Burundi; habcelestin@yahoo.fr; 4 Ecole Doctorale, Université du Burundi, Bujumbura 1550, Burundi; jushabani@yahoo.fr

**Keywords:** covariant Weyl-Heisenberg integral quantization, time-frequency, position-wave vector, space-time metric, general relativity, geometry of information, 46L65, 81S05, 81S30, 83C45, 81R30

## Abstract

As an extension of Gabor signal processing, the covariant Weyl-Heisenberg integral quantization is implemented to transform functions on the eight-dimensional phase space $\left(x,k\right)$ into Hilbertian operators. The $x=\left(x^{\mu }\right)$ values are space-time variables, and the $k=\left(k^{\mu }\right)$ values are their conjugate frequency-wave vector variables. The procedure is first applied to the variables $\left(x,k\right)$ and produces essentially canonically conjugate self-adjoint operators. It is next applied to the metric field $g_{\mu \nu }\left(x\right)$ of general relativity and yields regularized semi-classical phase space portraits ${\overset{ˇ}{g}}_{\mu \nu }\left(x\right)$. The latter give rise to modified tensor energy density. Examples are given with the uniformly accelerated reference system and the Schwarzschild metric. Interesting probabilistic aspects are discussed.

## 1. Introduction

One of the difficulties of accepting an arbitrary change in space-time coordinates in general relativity lies in the primordial learning of our environment. For instance, in the case of the Schwarzschild metric, $ds^{2}=\left(1-2m/r\right)dt^{2}-{\left(1-2m/r\right)}^{-1}dr^{2}-r^{2}d\theta^{2}-r^{2}\sin^{2}\theta d\phi^{2}$, the radial coordinate appears as the most natural and easily understandable in terms of basic physics, namely a mass and a radius, while numerous coordinates (e.g., Gullstrand-Painlevé, Kruskal-Szekeres, Lemaître, and Eddington-Finkelstein) used to examine the continuation of the Schwarzschild solution are not so immediate.

From the early stages of development, our senses perceive temporal and spatial dimensions of this environment. This training can be understood through the most basic physical laws, such as the conservation of energy, momentum, and angular momentum, through myriads of tiny collisions at all scales between any living matter and the rest of the world. In particular, the essentially Galilean perception of our tempo-spatial environment might be in mathematical terms compared with Fourier reconstruction and an analysis of tempo-spatial signals s(x),x:=(t,r) in Hilbert space $L^{2}\left(R^{4},d^{4}x=dt\;dr\right)\equiv H$.

(1) $$\begin{array}{cc} s(x) & =\frac{1}{4\pi^{2}}\int_{R^{4}}d^{4}k\;\hat{s}\left(k\right)\;e^{ik\cdot x}\;,\;k:=\left(\omega ,k\right)\;,\;k\cdot x:=\omega t-k\cdot r\;,\\ \hat{s}\left(k\right) & =\frac{1}{4\pi^{2}}\int_{R^{4}}d^{4}x\;s\left(x\right)\;e^{-ix\cdot k}\;. \end{array}$$

The minkowskian inner product k·x has been chosen for convenience; however, in the present context, the Fourier exponentials in the integrals (1) are not to be interpreted as a travelling wave. They are just the basic ingredients of the Fourier transform for determining the frequency ω and wave vector k content of the signal s(x). The latter have an inverse dimension of time and space vector, respectively, so no additional constants appear in the Fourier transform. Of course, they can appear in the expressions of the signals, and they can also be introduced into (1) to give more physical flavor to the above formula in terms of energy and momentum.

As is now well established in signal or image processing, Fourier techniques are advantageously replaced by Gabor reconstruction and analysis. One chooses a probe signal, or *window*, ψ, which is well localized in time-space and the frequency-wave vector at once, and which is normalized in H, ${∥\psi ∥}^{2}=\int_{R^{4}}d^{4}x\;{\left|\psi \left(x\right)\right|}^{2}=1$. This probe is then translated and modulated as

(2) $$\psi \left(x\right)\to \psi_{y,l}\left(x\right)=e^{il\cdot x}\;\psi \left(x-y\right)\;.$$

We then obtain the reconstruction of the signal s(x) in H in terms of its Gabor transform S(x,k):

(3) $$\begin{array}{cc} s(x) & =\frac{1}{{\left(2\pi \right)}^{4}}\int_{R^{8}}d^{4}y\;d^{4}l\;S\left(y,l\right)\;\psi_{y,l}\left(x\right),\\ S(x,k) & =\langle \psi_{x,k}|s\rangle =\int_{R^{4}}d^{4}y\;\bar{\psi_{x,k}\left(y\right)}\;s\left(y\right)\;. \end{array}$$

As is the case for Fourier, conservation of the energy (in the signal sense) holds:

(4) $$\int_{R^{4}}d^{4}{x\;|s\left(x\right)|}^{2}={∥s∥}^{2}=\frac{1}{{\left(2\pi \right)}^{4}}\int_{R^{8}}d^{4}x\;d^{4}{k\;|S\left(x,k\right)|}^{2}:={∥S∥}^{2}\;.$$

This *Plancherel* formula results from the resolution of the identity operator 𝟙 in H provided by the continuous non-orthogonal family of *coherent states*$\left\{\psi_{x,k}\;,\;\left(x,k\right)\in R^{8}\right\}$ (overcompleteness):

(5) $$𝟙=\frac{1}{{\left(2\pi \right)}^{4}}\int_{R^{8}}d^{4}x\;d^{4}k\;|\psi_{x,k}\rangle \langle \psi_{x,k}|\;.$$

Similar to the way in which the Fourier transform is based on the one-dimensional unitary irreducible representations (UIR) of the abelian translation group in $R^{4}$, the above Gabor transform is based on the infinite-dimensional von Neumann UIR of the Weyl-Heisenberg group $H_{4}$ [1]. This UIR is nothing more than the non-commutative extension of the translation group in $R^{8}$ when the latter is combined with a one-parameter cocycle. Quantum mechanics for one Galilean elementary system is itself based on the UIR of $H_{3}$.

In this article, we take advantage of the resolution of the identity in the Hilbert space of signals H to linearly map functions f(x,k) on the phase space $\left\{\left(x,k\right)\right\}∼R^{8}$ and, more generally, distributions [2] to operators $A_{f}$ in H. The procedure is called Weyl-Heisenberg covariant integral quantization, or more simply, Gabor quantization. According to the choice of the probe ψ, it can yield regularizations of the original *f* through spectral properties of the operator $A_{f}$ and expected values, *lower symbols*, or *semi-classical phase space portraits* $\overset{ˇ}{f}\left(x,k\right):=\langle \psi_{x,k}|A_{f}|\psi_{x,k}\rangle$ of $A_{f}$. The mathematical content of the present work is, of course, not original. It has been comprehensively developed over the past decades, with noticeable contributions by Werner [3], Feichtinger & Kozek [4], Luef & Skrettingland [5]. In this context, we should also mention the pioneer works by Klauder [6,7], Berezin [8], Daubechies & Grossmann [9,10] on the integral quantization based on the standard coherent state as particular cases of Gabor quantization with a quantum mechanics flavor. Concerning the genesis of specific aspects of our approach, see, for instance, the recent [11,12,13,14,15,16] for more explanations on the motivations and illustrations. The maps $f\mapsto A_{f}\mapsto \overset{ˇ}{f}$ have probabilistic features that have to be exploited within some informational interpretation and offer new perspectives, particularly in cosmology. Moreover, restricting the quantization to scalar fields g(x), such as the metric fields of general relativity, leads to interesting outcomes. Of course, the extension to tensorial fields is straightforward notwithstanding the increase in technicalities.

The two following sections are heavily inspired from the recent [15] and should be considered as a necessary reminder in view of extending our time-frequency ($R^{2}$) approach to the time-space frequency-wave vector $R^{8}$. Section 2 presents the integral quantization under its general form. In Section 3 and Section 4, we outline the Gabor quantization and its generalizations for the simplest case of the time-frequency phase space $\left\{\left(t,\omega \right)\right\}∼R^{2}$. In Section 5, we proceed with the analysis of the procedure in terms of observational and probabilistic aspects. In Section 6, we extend this material to the eight-dimensional case for which the symmetry group is $H_{4}$. However, for the sake of simplicity, we restrict our procedure to Gabor quantization based on the choice of probe functions ψ. In Section 7, motivated by the 1935 Einstein-Rosen paper [17], Gabor quantization is applied to the most elementary (and singular!) metric field of general relativity, namely, the uniformly accelerated reference system. We show how the regularizing constant (introduced by hand by the authors) naturally emerges from our procedure. In Section 8, we also consider another historical metric, the Schwarzschild solution, and show how Gabor quantization and its phase space portraits lead to appealing regularizations, at the price of breaking the rotational symmetry. In Section 9, we end the article with some interesting perspectives.

## 2. From Signal Analysis and Reconstruction to Integral Quantization

Let (X,μ) be a measure space and H be a (separable) Hilbert space. An operator-valued function

(6) X∋x↦M(x)actinginH

resolves the identity operator 𝟙 in H with respect to the measure μ if

(7) $$\int_{X}M\left(x\right)\;d\mu \left(x\right)=𝟙$$

holds in a weak sense, i.e., $\int_{X}\langle \psi |M\left(x\right)|\psi^{\prime }\rangle \;d\mu \left(x\right)=\langle \psi |\psi^{\prime }\rangle$ for any $\psi ,\psi^{\prime }$ in the common domain of the M(x) values, a.e. *x*.

As we have in (3), the *analysis* and *reconstruction* of a signal are described by the action of (7) on a vector in H

(8) $$H∋|s\rangle \overset{\mathrm{reconstruction}}{=}\int_{X}\overset{\mathrm{analysis}}{\overbrace{M(x)|s\rangle }}\;d\mu \left(x\right)\;.$$

On the other hand, *integral quantization* is the linear map of a function on *X* to an operator in H, which is defined by

(9) $$f\left(x\right)\mapsto \int_{X}f\left(x\right)M\left(x\right)\;d\mu \left(x\right)=A_{f}\;,\;1\mapsto 𝟙\;.$$

If the operators M(x) in (7) are nonnegative and bounded, i.e., 〈ϕ|M(x)|ϕ〉≥0 for (almost) all x∈X and all ϕ∈H, one says that they form a (normalized) positive operator-valued measure (POVM) on *X*. If they are a further unit trace-class, i.e., tr(M(x))=1 for all x∈X, i.e., if the M(x) values are density operators, then the map

(10) $$f\left(x\right)\mapsto \overset{ˇ}{f}\left(x\right):=\mathrm{tr}\left(M\left(x\right)A_{f}\right)=\int_{X}f\left(x^{\prime }\right)\;\mathrm{tr}\left(M\left(x\right)M\left(x^{\prime }\right)\right)\;d\mu \left(x^{\prime }\right)$$

is a local averaging of the original f(x) (which can be very singular, like a Dirac!) with respect to the probability distribution on *X*,

(11) $$x^{\prime }\mapsto \mathrm{tr}\left(M\left(x\right)M\left(x^{\prime }\right)\right)\;.$$

This averaging, or semi-classical portrait of the operator $A_{f}$, is in general a regularization, depending of course on the topological nature of the measure space (X,μ) and the functional properties of the M(x) values.

Now, consider a set of parameters κ and corresponding families of POVM $M_{\kappa }\left(x\right)$ solving the identity

(12) $$\int_{X}M_{\kappa }\left(x\right)\;d\mu \left(x\right)=𝟙\;.$$

One says that the *classical limit* f(x) holds at $\kappa_{0}$ if

(13) $${\overset{ˇ}{f}}_{\kappa }\left(x\right):=\int_{X}f\left(x^{\prime }\right)\;\mathrm{tr}\left(M_{\kappa }\left(x\right)M_{\kappa }\left(x^{\prime }\right)\right)\;d\mu \left(x^{\prime }\right)\to f\left(x\right)\;as\;\kappa \to \kappa_{0}\;,$$

where the convergence $\overset{ˇ}{f}\to f$ is defined in the sense of a certain topology.

Otherwise, $\mathrm{tr}(M_{\kappa }\left(x\right)M_{\kappa }\left(x^{\prime }\right))$ tends to

(14) $$\mathrm{tr}\left(M_{\kappa }\left(x\right)M_{\kappa }\left(x^{\prime }\right)\right)\to \delta_{x}\left(x^{\prime }\right)$$

where $\delta_{x}$ is a Dirac measure with respect to μ,

(15) $$\int_{X}f\left(x^{\prime }\right)\;\delta_{x}\left(x^{\prime }\right)\;d\mu \left(x^{\prime }\right)=f\left(x\right)\;.$$

In fact, nothing guarantees the existence of such a limit on a general level. Nevertheless, if the semi-classical ${\overset{ˇ}{f}}_{\kappa }$ might appear as more realistic and more easily handled than the original *f*, the range of acceptability of the parameters κ needs to be evaluated.

## 3. Gabor Quantization of Functions of Time-Frequency Variables

In this section, we present the material in the basic two-dimensional time-frequency case in order to progressively familiarize the reader with our quantization method.

### 3.1. Gabor Quantization

In order to manage time-frequency functions f(b,ω), we first start from the resolution of the identity provided by the Gabor POVM, which is built as follows.

(16) $$𝟙=\int_{R^{2}}\frac{db\;d\omega }{2\pi }|\psi_{b\omega }\rangle \langle \psi_{b\omega }|\;,\;\langle \psi_{b\omega }|\psi_{b^{\prime }\omega^{\prime }}\rangle \neq \delta \left(b-b^{\prime }\right)\;\delta \left(\omega -\omega^{\prime }\right)$$

where the functions

(17) $$\langle \delta_{t}|\psi_{b\omega }\rangle :=e^{i\omega t}\psi \left(t-b\right)$$

are the modulated-transported unit-norm probe-vectors in $L^{2}\left(R,dt\right)$ and where $R⊃\Delta \mapsto \int_{\Delta }\frac{db\;d\omega }{2\pi }|\psi_{b\omega }\rangle \langle \psi_{b\omega }|$ is the corresponding normalized POVM on the plane. From (9), the quantization of f(b,ω) is given by

(18) $$f\mapsto A_{f}=\int_{R^{2}}\frac{db\;d\omega }{2\pi }\;f\left(b,\omega \right)\;|\psi_{b\omega }\rangle \langle \psi_{b\omega }|\;.$$

The latter maps a function (or tempered distribution) f(b,ω) on the time-frequency plane to the integral operator $A_{f}$ acting in the Hilbert space of finite-energy signals as

(19) $$\left(A_{f}s\right)\left(t\right)=\langle \delta_{t}|A_{f}|s\rangle =\int_{-\infty }^{+\infty }dt^{\prime }\;A_{f}\left(t,t^{\prime }\right)\;s\left(t^{\prime }\right)\;,$$

with the integral kernel given by

(20) $$A_{f}\left(t,t^{\prime }\right)=\frac{1}{\sqrt{2\pi }}\int_{-\infty }^{+\infty }db\;{\hat{f}}_{\omega }\left(b,t^{\prime }-t\right)\;\psi \left(t-b\right)\;\bar{\psi (t^{\prime }-b)}.$$

Here ${\hat{f}}_{\omega }\left(b,y\right)$ is the partial Fourier transform with respect to the variable ω:

(21) $${\hat{f}}_{\omega }\left(b,y\right)=\frac{1}{\sqrt{2\pi }}\int_{-\infty }^{+\infty }d\omega \;f\left(b,\omega \right)\;e^{-i\omega y}\;.$$

The outcome of the construction of the operator $A_{f}$ through (19) should be analyzed from the quantum measurement viewpoint. Within the framework of quantum physics, a physically relevant operator of the above form $A_{f}$ is a self-adjoint operator whose expectation value is the “unsharp” representation [18,19,20]

(22) $$\begin{array}{c} \mathrm{tr}\left(\rho_{m}A_{f}\right)=\int_{X}f\left(x\right)\;\mathrm{tr}\left(\rho_{m}\rho \left(x\right)\right)\;d\nu \left(x\right)\;, \end{array}$$

where $\rho_{m}$ is a density operator (∼mixed quantum state) describing the way the physical system under observation has been prepared. In (18), a real function f(b,ω) is mapped to a symmetric operator, and if *f* is semi-bounded, then $A_{f}$ is self-adjoint through the Friedrichs extension [21] of its associated semi-bounded quadratic form. For an unbounded real *f*, self-adjointness is not guaranteed and should be individually investigated.

Let us go through some specific situations. The Gabor quantization of separable functions f(b,ω)=u(b)v(ω) yields the integral kernel

(23) $$A_{uv}\left(t,t^{\prime }\right)=\frac{\hat{v}\left(t^{\prime }-t\right)}{\sqrt{2\pi }}\int_{-\infty }^{+\infty }db\;u\left(b\right)\;\psi \left(t-b\right)\;\bar{\psi (t^{\prime }-b)}\;,$$

where $\hat{v}$ is the Fourier transform of *v*. Therefore, the action on a signal s(t) reads as the combination of convolution and multiplication

(24) $$\left(A_{uv}s\right)\left(t\right)=\frac{1}{\sqrt{2\pi }}\int_{-\infty }^{+\infty }db\;\psi \left(b\right)\;u\left(t-b\right)\;\left({\bar{\tilde{\psi }}}_{b}\hat{\tilde{v}}*s\right)\left(t\right)\;,\;\psi_{b}\left(t\right):=\psi \left(t-b\right)\;,$$

where $\tilde{\psi }\left(t\right):=\psi \left(-t\right)$. In the monovariable case f(b,ω)=u(b), the integral kernel (20) reads as

(25) $$A_{u}\left(t,t^{\prime }\right)=\delta \left(t^{\prime }-t\right)\int_{-\infty }^{+\infty }db\;u\left(b\right)\;{\left|\psi \left(t-b\right)\right|}^{2}=\delta \left(t^{\prime }-t\right)\;\left({\left|\psi \right|}^{2}*u\right)\left(t\right)\;,$$

and one obtains the multiplication operator

(26) $$\left(A_{u(b)}s\right)\left(t\right)=\left(u*{\left|\psi \right|}^{2}\right)\left(t\right)\;s\left(t\right)\;.$$

For the other monovariable case f(b,ω)=v(ω), one obtains the integral kernel

(27) $$A_{v(\omega )}\left(t,t^{\prime }\right)=\frac{\hat{v}\left(t^{\prime }-t\right)}{\sqrt{2\pi }}\int_{-\infty }^{+\infty }db\;\psi \left(b\right)\;\bar{\tilde{\psi }}\left(t-t^{\prime }-b\right)=\frac{\hat{v}\left(t^{\prime }-t\right)}{\sqrt{2\pi }}\;R_{\psi \psi }\left(t-t^{\prime }\right)$$

where

(28) $$R_{\psi \psi }\left(t\right):=\int_{-\infty }^{+\infty }dt^{\prime }\;\psi \left(t^{\prime }\right)\;\bar{\psi (t^{\prime }-t)}=\left(\psi *\bar{\tilde{\psi }}\right)\left(t\right)={\bar{\tilde{R}}}_{\psi \psi }\left(t\right)$$

is the autocorrelation of the probe, i.e., the correlation of the probe with a delayed copy of itself as a function of delay. Note that

(29) $$\frac{1}{\sqrt{2\pi }}R_{\psi \psi }\left(t\right)=F^{-1}\left[|\hat{\psi }{|}^{2}\right]\left(t\right)\;.$$

We eventually obtain the convolution operator acting on the signal:

(30) $$\left(A_{v(\omega )}s\right)\left(t\right)=\frac{1}{\sqrt{2\pi }}\left[\left(R_{\psi \psi }\hat{\tilde{v}}\right)*s\right]\left(t\right).$$

The application of these formulas to time and frequency variables yields the centered time and frequency operators:

(31) $$\begin{array}{cc} A_{b} & =T-{\langle T\rangle }_{\psi }\;𝟙\;,\;\left(Ts\right)\left(t\right)=ts\left(t\right)\;, \end{array}$$

(32) $$\begin{array}{cc} A_{\omega } & =\Omega -{\langle \Omega \rangle }_{\psi }\;𝟙\;,\;\left(\Omega s\right)\left(t\right)=-i\;\partial_{t}s\left(t\right)\;, \end{array}$$

where ${\langle A\rangle }_{\psi }$ is the mean value of the operator *A* in the state ψ, as it is usually denoted within the quantum framework. The choice of an even probe ψ allows one to eliminate these constants. It is the case with the standard choice of the normalized centred Gaussian with width σ

(33) $$\psi \left(t\right)\equiv G_{\sigma }\left(t\right)=\frac{1}{\pi^{1/4}\sqrt{\sigma }}e^{-\frac{t^{2}}{2\sigma^{2}}}\;.$$

Note that its autocorrelation is also a (non-normalized) Gaussian:

(34) $$R_{G_{\sigma }G_{\sigma }}\left(t\right)=e^{-\frac{t^{2}}{4\sigma^{2}}}\;.$$

The operators *T* and Ω, like $A_{b}$ and $A_{\omega }$, are essentially self-adjoint and obey the “canonical” commutation rule (no *ℏ* here!):

(35) TΩ−ΩT≡[T,Ω]=i𝟙,

with its immediate Fourier uncertainty consequence

(36) $$\Delta_{s}T\;\Delta_{s}\Omega \geq \frac{1}{2}\;,\;\Delta_{s}A:=\sqrt{\langle s|A^{2}|s\rangle -{\left(\langle s|A|s\rangle \right)}^{2}}.$$

Now, one should keep in mind that the CCR [A,B]=i𝟙 for a self-adjoint (*A*, *B*) pair, with a common domain, holds true only if both have a continuous spectrum (−∞,+∞). The expression of the CCR in terms of the respective unitary operators reads as

(37) $$e^{i\sigma \Omega }\;e^{i\tau T}=e^{i\sigma \tau }e^{i\tau T}\;e^{i\sigma \Omega }\;,\;\left(\mathrm{Weyl}\mathrm{relations}\right)\;.$$

von Neumann proved (1931) that, up to multiplicity and unitary equivalence, the Weyl relations have only one solution (see [22] for the proof). In the present formalism, time is elevated to the status of a quantum observable. This is absolutely not the case for the time operator of quantum mechanics (see, for instance, the review [23]). Indeed, in quantum mechanics, the Hamiltonian is bounded below and so the quantum time, although symmetric, is not self-adjoint and has no self-adjoint extension.

Concerning the square of time and frequency variables, we obtain:

(38) $$\begin{array}{cc} A_{b^{2}} & ={\left(T-{\langle T\rangle }_{\psi }\right)}^{2}+\Delta_{\psi }^{2}T\;𝟙\;, \end{array}$$

(39) $$\begin{array}{cc} A_{\omega^{2}} & ={\left(\Omega -{\langle \Omega \rangle }_{\psi }\right)}^{2}+\Delta_{\psi }^{2}\Omega \;𝟙\;. \end{array}$$

With the choice of Gaussian probe (33), these operators read

(40) $$A_{b^{2}}=T^{2}+\frac{\sigma^{2}}{2}\;𝟙\;,\;A_{\omega^{2}}=\Omega^{2}+\frac{\sigma^{2}}{2}\;𝟙\;.$$

### 3.2. Gabor Semi-Classical Portrait

The Gabor semi-classical phase-space portrait of $A_{f}$ is given by

(41) $$\overset{ˇ}{f}\left(b,\omega \right)=\int_{R^{2}}\frac{db^{\prime }\;d\omega^{\prime }}{2\pi }\;f\left(b^{\prime },\omega^{\prime }\right)\;{\left|\langle \psi_{b\omega }|\psi_{b^{\prime }\omega^{\prime }}\rangle \right|}^{2}\;.$$

The overlap $\langle \psi_{b\omega }|\psi_{b^{\prime }\omega^{\prime }}\rangle$ is given by

(42) $$\begin{array}{cc} \langle \psi_{b\omega }|\psi_{b^{\prime }\omega^{\prime }}\rangle & =\int_{R}dt\;e^{-i(\omega^{\prime }-\omega )t}\;\bar{\psi (t-b^{\prime })}\;\psi \left(t-b\right)\\ \; & =e^{-i(\omega^{\prime }-\omega )b}\left(F\left[t_{b^{\prime }-b}\bar{\psi }\right]*F\left[\psi \right]\right)\left(\omega^{\prime }-\omega \right)\;, \end{array}$$

where $F\left[f\right]:=\hat{f}$ (Fourier transform), $\left(t_{b}f\right)\left(t\right):=f\left(t-b\right)$, and

$$\left(b^{\prime },\omega^{\prime }\right)\mapsto {\left|\langle \psi_{b\omega }|\psi_{b^{\prime }\omega^{\prime }}\rangle \right|}^{2}/2\pi$$

is a probability distribution on the phase space equipped with the Lebesgue measure $db^{\prime }\;d\omega^{\prime }$. Hence, the regularity properties of $\overset{ˇ}{f}$ depend on those of the probe. The function $\overset{ˇ}{f}\left(b,\omega \right)$ is an elaborate combination of a partial Fourier transform and multi-convolutions:

(43) $$\overset{ˇ}{f}\left(b,\omega \right)=\frac{1}{\sqrt{2\pi }}\int_{R}d\tau \;e^{i\omega \tau }\int_{R}db^{\prime }\;{\hat{f}}_{\omega }\left(b-b^{\prime },\tau \right)\;\left(\left(\bar{\tilde{\psi }}\;\tilde{t_{b^{\prime }}\psi }\right)*\left(\bar{t_{-b^{\prime }}\psi }\;\psi \right)\right)\left(\tau \right)\;.$$

In the monovariable case f(b,ω)=u(b), this formula simplifies to

(44) $$\overset{ˇ}{u}\left(b,\omega \right)\equiv \overset{ˇ}{u}\left(b\right)=\left(u*\left({\left|\psi \right|}^{2}*{\left|\tilde{\psi }\right|}^{2}\right)\right)\left(b\right)=\left(u*R_{{\left|\psi \right|}^{2}{\left|\psi \right|}^{2}}\right)\left(b\right)\;$$

where we note the appearance of the autocorrelation of the probability distribution $t\mapsto {\left|\psi \left(t\right)\right|}^{2}$ on the temporal axis. For the simplest cases, time and time squared, we obtain

(45) $$\overset{ˇ}{b}=b-{\langle b\rangle }_{R_{{\left|\psi \right|}^{2}{\left|\psi \right|}^{2}}}\;,\;\overset{ˇ}{b^{2}}={\left(b-{\langle b\rangle }_{R_{{\left|\psi \right|}^{2}{\left|\psi \right|}^{2}}}\right)}^{2}+\sigma_{R_{{\left|\psi \right|}^{2}{\left|\psi \right|}^{2}}}^{2}\left(b\right)\;,$$

where ${\langle s\rangle }_{p}$ stands for the expected value of the signal *s* with respect to the probability distribution *p*, and $\sigma_{p}^{2}\left(s\left(t\right)\right)$ is its variance with respect to *p*. An analogous formula holds (in the Fourier side) for the monovariable case f(b,ω)=v(ω):

(46) $$\overset{ˇ}{v}\left(b,\omega \right)\equiv \overset{ˇ}{v}\left(\omega \right)=\bar{F}\left[\hat{v}{\left(\bar{\tilde{\psi }}*\psi \right)}^{2}\right]\left(\omega \right)\;,\;\bar{F}:=F^{-1}\;.$$

With the choice of Gaussian probe (33), the overlap (42) is given by

(47) $$\langle G_{\sigma ,b^{\prime },\omega^{\prime }}|G_{\sigma ,b,\omega }\rangle =e^{-i\left(\omega^{\prime }-\omega \right)\frac{b+b^{\prime }}{2}}\;e^{-\frac{{\left(b-b^{\prime }\right)}^{2}}{4\sigma^{2}}}\;e^{-\frac{\sigma^{2}{\left(\omega -\omega^{\prime }\right)}^{2}}{4}}\;,$$

and the semi-classical portrait of the operator $A_{f}$ is the double Gaussian convolution:

(48) $$\overset{ˇ}{f}\left(b,\omega \right)=\int_{R^{2}}\frac{db^{\prime }\;d\omega^{\prime }}{2\pi }\;f\left(b^{\prime },\omega^{\prime }\right)\;e^{-\frac{{\left(b-b^{\prime }\right)}^{2}}{2\sigma^{2}}}\;e^{-\frac{\sigma^{2}{\left(\omega -\omega^{\prime }\right)}^{2}}{2}}\;.$$

As a consequence, we observe that no classical limit holds at σ→0 or σ→∞. This is just an illustration of the time-frequency uncertainty principle. Separable functions f(b,ω)=u(b)v(ω) remain separable. From Equation (44) and the convolution of two Gaussians, one finds

(49) $$\overset{ˇ}{f}\left(b,\omega \right)=\left(u*G_{\sqrt{2}\sigma }^{2}\right)\left(b\right)\;\left(v*G_{\sqrt{2}/\sigma }^{2}\right)\left(\omega \right)\;,$$

and, in particular

(50) $$\overset{ˇ}{u}\left(b\right)=\left(u*G_{\sqrt{2}\sigma }^{2}\right)\left(b\right)\;,\;\overset{ˇ}{v}\left(\omega \right)=\left(v*G_{\sqrt{2}/\sigma }^{2}\right)\left(\omega \right)\;.$$

Here, the classical limit exists separately, and the convergence is simple at least for a sufficiently regular *u* and *v*:

(51) $$\overset{ˇ}{u}\left(b\right)\underset{\sigma \to 0}{\to }u\left(b\right)\;,\;\overset{ˇ}{v}\left(\omega \right)\underset{\sigma \to \infty }{\to }v\left(\omega \right)\;.$$

For the simplest cases,

(52) $$\begin{array}{cc} \overset{ˇ}{b} & =b\;,\;\overset{ˇ}{b^{2}}=b^{2}+\sigma^{2}\;, \end{array}$$

(53) $$\begin{array}{cc} \overset{ˇ}{\omega } & =\omega \;,\;\overset{ˇ}{\omega^{2}}=\omega^{2}+\frac{1}{\sigma^{2}}\;. \end{array}$$

## 4. Beyond Gabor: The Weyl-Heisenberg Covariant Integral Quantization of the Time-Frequency Plane

Gabor signal analysis and quantization are the simplest ones among a vast amount of possibilities, all of them being based on the unitary dual of the Weyl-Heisenberg group. We remind the reader here of the most important features of this group that we use in our approach to quantization. More details are given in the pedagogical [11].

We recognize in the construction of the Gabor family (2) the combined actions of the two unitary operators introduced in (37), with the respective generators of the self-adjoint time and the frequency operators

(54) $$L^{2}\left(R,dt\right)∋\psi \left(t\right)\mapsto \psi_{b,\omega }\left(t\right)=\left(e^{i\omega T}e^{-ib\Omega }\psi \right)\left(t\right)\;.$$

Two alternative forms of the action (54) are provided by the Weyl formulas (37) combined with the Baker-Campbell-Hausdorff formula:

(55) $$\psi_{b,\omega }\left(t\right)=e^{ib\omega }\left(e^{-ib\Omega }e^{i\omega T}\psi \right)\left(t\right)=e^{i\frac{b\omega }{2}}\left(e^{i(\omega T-b\Omega )}\psi \right)\left(t\right)\;.$$

In the above, the Weyl or displacement operator appears up to a phase factor:

(56) $$e^{i(\omega T-b\Omega )}=D\left(b,\omega \right)\;,\;\psi_{b,\omega }\left(t\right)=e^{i\frac{b\omega }{2}}\left(D(b,\omega )\psi \right)\left(t\right)\;.$$

The appearance of this phase factor, like that appearing in the composition formula (37), indicates that the map (b,ω)↦D(b,ω) is a *projective* unitary representation of the time-frequency abelian plane. Dealing with a true representation necessitates the introduction of a third degree of freedom to account for this phase factor. Hence, we work with the Weyl-Heisenberg group $G_{\mathrm{WH}}$.

(57) $$G_{\mathrm{WH}}=\left\{g=\left(\varsigma ,b,\omega \right)\;,\;\varsigma \in R\;,\;\left(b,\omega \right)\in R^{2}\right\}\;,$$

with neutral element (0,0,0), and

(58) $$g_{1}g_{2}=\left(\varsigma_{1}+\varsigma_{2}+\frac{1}{2}\left(\omega_{1}b_{2}-\omega_{2}b_{1}\right)\;,b_{1}+b_{2},\;\omega_{1}+\omega_{2}\right)\;,\;g^{-1}=\left(-\varsigma ,-b,-\omega \right)\;.$$

The Weyl-Heisenberg group symmetry underlying the Gabor transform is understood through its unitary irreducible representation (UIR). As a result of the von Neumann uniqueness theorem, any infinite-dimensional UIR, *U*, of $G_{\mathrm{WH}}$ is characterized by a real number λ≠0 (there is also the degenerate, one-dimensional UIR corresponding to λ=0). If the Hilbert space carrying the UIR is the space of finite-energy signals $H=L^{2}\left(R,dt\right)$, the representation operators are defined by the action similar to (54) (with the choice λ=1) and completed with a phase factor:

(59) $$U\left(\varsigma ,b,\omega \right)=e^{i\varsigma }e^{-i\omega b/2}\;e^{i\omega T}\;e^{-ib\Omega }=e^{i\varsigma }\;D\left(b,\omega \right)\;.$$

We now pick a bounded trace-class operator $Q_{0}$ on H with

(60) $$\mathrm{tr}\;Q_{0}=1\;.$$

Its unitary Weyl-Heisenberg transport yields the continuous family of bounded trace-class operators

(61) $$Q\left(b,\omega \right)=U\left(\varsigma ,b,\omega \right)Q_{0}U{\left(\varsigma ,b,\omega \right)}^{†}=D\left(b,\omega \right)Q_{0}D{\left(b,\omega \right)}^{†}\;.$$

Applying the Schur Lemma to the irreducible projective unitary representation (b,ω)↦D(b,ω) allows one to prove the resolution of the identity obeyed by the operator-valued function Q(b,ω) on the time-frequency plane:

(62) $$\int_{R^{2}}Q\left(b,\omega \right)\;\frac{db\;d\omega }{2\pi }=𝟙\;.$$

The Weyl-Heisenberg integral quantization in its most general formulation ensues:

(63) $$f\left(b,\omega \right)\mapsto A_{f}=\int_{R^{2}}f\left(b,\omega \right)\;Q\left(b,\omega \right)\;\frac{db\;d\omega }{2\pi }\;.$$

If $Q_{0}$ is symmetric, a real function f(b,ω) is mapped to a symmetric operator $A_{f}$. Moreover, if $Q_{0}$ is non-negative, i.e., a density operator, then a real semi-bounded function f(b,ω) is mapped to a self-adjoint operator $A_{f}$, as was been stated about Gabor quantization (19).

Due to the homogeneity of the phase space (i.e., the choice of the origin is arbitrary), we have translational covariance in the sense that the quantization of the translated *f* is unitarily equivalent to the quantization of *f*:

(64) $$\begin{array}{cc} \; & U\left(\varsigma ,b_{0},\omega_{0}\right)\;A_{f}\;U{\left(\varsigma ,b_{0},\omega_{0}\right)}^{†}=A_{T(b_{0},\omega_{0})f}\;,\\ & \mathrm{where}\;\left(T(b_{0},\omega_{0})f\right)\left(b,\omega \right):=f\left(b-b_{0},\omega -\omega_{0}\right)\;. \end{array}$$

Note that the Gabor quantization (18) corresponds to the choice of the orthogonal projector $Q_{0}=\left|\psi \rangle \langle \psi \right|$.

There is a form that is equivalent to the quantization (63). This form is expressed in terms of the displacement operator D, the symplectic Fourier transform of f(b,ω),

(65) $$f_{s}\left[f\right]\left(b,\omega \right):=\int_{R^{2}}e^{-i(b\omega^{\prime }-b^{\prime }\omega )}\;f\left(b^{\prime },\omega^{\prime }\right)\;\frac{db^{\prime }\;b\omega^{\prime }}{2\pi }\;,\;f_{s}^{2}=𝟙\;,$$

and the Weyl transform of the operator $Q_{0}$ defined as the “apodization” function on the time-frequency plane:

(66) $$\Pi \left(b,\omega \right):=\mathrm{tr}\left(D\left(-b,-\omega \right)Q_{0}\right)\;,\;\Pi \left(0,0\right)=1\;.$$

The inverse of the latter transform is given by

(67) $$Q_{0}=\int_{R^{2}}D\left(b,\omega \right)\;\Pi \left(b,\omega \right)\;\frac{db\;d\omega }{2\pi }\;,$$

which means that $Q_{0}$ is the quantization of f(b,ω)=2πδ(b,ω) such that $\bar{f_{s}}\left[f\right]\left(b,\omega \right)=1$.

One can then obtain

(68) $$A_{f}=\int_{R^{2}}D\left(b,\omega \right)\;\bar{f_{s}}\left[f\right]\left(b,\omega \right)\;\Pi \left(b,\omega \right)\;\frac{db\;d\omega }{2\pi }\;.$$

From this form, the action $A_{f}$ is obtained as the integral operator:

(69) $$L^{2}\left(R,dt\right)∋s\left(t\right)\mapsto \left(A_{f}s\right)\left(t\right)=\int_{-\infty }^{+\infty }dt^{\prime }\;A_{f}\left(t,t^{\prime }\right)\;s\left(t^{\prime }\right)\;,$$

with the kernel given by

(70) $$A_{f}\left(t,t^{\prime }\right)=\frac{1}{2\pi }\int_{-\infty }^{+\infty }db\;{\hat{f}}_{\omega }\left(b,t^{\prime }-t\right)\;{\hat{\Pi }}_{\omega }\left(t-t^{\prime },b-\frac{t+t^{\prime }}{2}\right)\;,$$

where the partial Fourier transforms ${\hat{f}}_{\omega }$ and ${\hat{\Pi }}_{\omega }$ are defined in (21).

The semi-classical portrait (10) of $A_{f}$ now reads

(71) $$\overset{ˇ}{f}\left(b,\omega \right)=\mathrm{tr}\left(Q\left(b,\omega \right)A_{f}\right)=\int_{R^{2}}f\left(b^{\prime },\omega^{\prime }\right)\;\mathrm{tr}\left(Q\left(b,\omega \right)Q\left(b^{\prime },\omega^{\prime }\right)\right)\;\frac{db^{\prime }\;d\omega^{\prime }}{2\pi }\;.$$

Equivalently,

(72) $$\begin{array}{cc} \overset{ˇ}{f}\left(b,\omega \right) & =\int_{R^{2}}f_{s}\left[\Pi \;\tilde{\Pi }\right]\left(b-b^{\prime },\omega -\omega^{\prime }\right)\;f\left(b^{\prime },\omega^{\prime }\right)\;\frac{db^{\prime }\;d\omega^{\prime }}{2\pi }\\ \; & =\int_{R^{2}}f_{s}\left[\Pi \right]*f_{s}\left[\tilde{\Pi }\right]\left(b-b^{\prime },\omega -\omega^{\prime }\right)\;f\left(b^{\prime },\omega^{\prime }\right)\;\frac{db^{\prime }\;d\omega^{\prime }}{4\pi^{2}}\;, \end{array}$$

where $\tilde{\Pi }\left(b,\omega \right)=\Pi \left(-b,-\omega \right)$.

## 5. Probabilistic Aspects

Let us develop the probabilistic content of the quantization and smoothing procedure described in the previous section.

With a true probabilistic content, i.e., with a choice of operator $Q_{0}$, or, equivalently, with a choice of function Π in (66), such that

(73) $$\mathrm{tr}\left(Q\left(b,\omega \right)Q\left(b^{\prime },\omega^{\prime }\right)\right)\geq 0\;\forall \;\left(b,\omega \right)\;\mathrm{and}\;\left(b^{\prime },\omega^{\prime }\right)\;a.e.\;,$$

the meaning of the convolution appearing in the integral (72)

(74) $$\frac{1}{2\pi }f_{s}\left[\Pi \right]*f_{s}\left[\tilde{\Pi }\right]$$

is clear: it is the probability distribution (with respect to the measure dbdω/2π) for the difference of two vectors in the time-frequency plane, viewed as independent random variables, and thus is adapted to the abelian and homogeneous structure of the latter (the choice of origin is arbitrary!).

Let us examine in more detail the relationship between $Q_{0}$ and its Weyl transform Π. First, we note the important property issued from (66). If $Q_{0}$ is bounded symmetric, i.e., self-adjoint, then its Weyl transform is “CP” symmetric:

(75) $$\bar{\Pi (b,\omega )}=\Pi \left(-b,-\omega \right)\;.$$

Second, due to the unit trace condition on $Q_{0}$, the symplectic Fourier transform of Π is a quasi-probability distribution,

(76) $$\int_{R^{2}}f_{s}\left[\Pi \right]\left(b,\omega \right)\frac{db\;d\omega }{2\pi }=1\;.$$

The trivial case corresponds to Π(b,ω)=1, of course. Thus, $f_{s}\left[\Pi \right]\left(b,\omega \right)=2\pi \delta \left(b,\omega \right)$ is a probability distribution, like its squared convolution (73), despite the fact that $Q_{0}$ is not a density operator, $Q_{0}=2P$, where P is the parity operator (whose trace can be consistently defined as tr(P)=1/2). This choice for no filtering yields the popular Weyl–Wigner integral quantization equivalent to the standard (∼canonical) quantization.

In the case of Gabor quantization with a probe ψ, we have already established in (41) that (74) is equal to $|\langle \psi_{b\omega }|\psi_{b^{\prime }\omega^{\prime }}\rangle {|}^{2}$. The corresponding $\Pi_{\psi }$ is given by

(77) $$\begin{array}{cc} \Pi_{\psi }\left(b,\omega \right) & =\langle \psi |D(-b,-\omega )\psi \rangle =\langle D(b,\omega )\psi |\psi \rangle \\ & =\sqrt{2\pi }e^{-i\frac{b\omega }{2}}\left(F\left[\bar{\psi }\right]*F\left[t_{-b}\psi \right]\right)\left(\omega \right)\;, \end{array}$$

where F is the ordinary Fourier transform, and $F\left[t_{-b}\psi \right]\left(\omega \right)=e^{ib\omega }F\left[\psi \right]\left(\omega \right)$.

We are especially interested in building a self-adjoint $A_{f}$ from physically relevant real functions or distributions f(b,ω) through (63) that also yield the probability distribution (74). An appealing reason for the operator $Q_{0}$ is that it belongs to the class of density operators for which (73) holds true, or, equivalently, that the quantity $f_{s}\left[\Pi \right]*f_{s}\left[\tilde{\Pi }\right]/2\pi$, built from the corresponding Π, is a probability distribution on the time-frequency plane equipped with the measure dbdω/2π. The latter form of the condition is important because there is no guarantee that $f_{s}\left[\Pi \right]$ itself is positive and thus defines a probability distribution. For instance, in the Gabor case, $f_{s}\left[\Pi_{\psi }\right]$ is equal to

(78) $$\begin{array}{cc} f_{s}\left[\Pi \right]\left(b,\omega \right) & =\int_{-\infty }^{\infty }dt\;e^{-i\omega t}\;\bar{\psi \left(\frac{t}{2}-b\right)}\;\psi \left(-\frac{t}{2}-b\right)\\ & \equiv 2\pi W_{\psi }\left(-b,-\omega \right)\;, \end{array}$$

where $W_{\psi }$ is the Wigner function [24,25] of ψ, and it is well known that the latter is a probability distribution only if ψ(t) has the form

(79) ψ(t)=Cste−at2+bt+c,a,b,c∈C,Re(a)>0.

Let us now examine the standard Gaussian examples of functions $\Pi ={\tilde{\Pi }}_{G}$ having a probabilistic symplectic Fourier transform

(80) $$\begin{array}{cc} \Pi_{G}\left(b,\omega \right)=e^{-\frac{b^{2}}{2\sigma^{2}}}e^{-\frac{\omega^{2}}{2\tau^{2}}}\;, & \;f_{s}\left[\Pi_{G}\right]\left(b,\omega \right)=\sigma \tau e^{-\frac{\tau^{2}b^{2}}{2}}e^{-\frac{\sigma^{2}\omega^{2}}{2}}\\ f_{s}{\left[\Pi_{G}\right]}^{*2}\left(b,\omega \right) & =\frac{\sigma \tau }{2}e^{-\frac{\tau^{2}b^{2}}{4}}e^{-\frac{\sigma^{2}\omega^{2}}{4}}\;. \end{array}$$

Depending on the pair (σ,τ), the above functions do or do not yield non-negative operators $Q_{0}$. They do if $\sigma =\tau :=\sqrt{2\tanh\frac{1}{\Theta }}<\sqrt{2}$. We then obtain the Boltzman–Planck-like density operator [2]:

(81) $$Q_{0}=\left(1-e^{-\frac{1}{\Theta }}\right)\sum_{n=0}^{\infty }e^{-\frac{n}{\Theta }}\left|n\rangle \langle n\right|\equiv \rho \left(\Theta \right)\;,$$

in the Hilbertian basis made of eigenvectors |n〉 of the harmonic operator

(82) $$\left(\frac{\Omega^{2}}{2\omega_{0}^{2}}+\frac{T^{2}}{2t_{0}^{2}}\right)|n\rangle =\frac{1}{\omega_{0}t_{0}}\left(n+\frac{1}{2}\right)|n\rangle \;.$$

For convenience, one chooses units $\omega_{0}=1=t_{0}$.

In this context, the parameter Θ plays the role of temperature (in suitable units). More generally, from the inverse Weyl transform (67), we obtain the matrix elements of $Q_{0}$ in the above basis {|n〉} in terms of those of the displacement operator:

(83) $${\left(Q_{0}\right)}_{mn}:=\langle m|Q_{0}|n\rangle =\int_{R^{2}}D_{mn}\left(b,\omega \right)\;\Pi \left(b,\omega \right)\;\frac{db\;d\omega }{2\pi }\;.$$

The latter involves associated Laguerre polynomials $L_{n}^{\left(\alpha \right)}\left(t\right)$ [26]:

(84) $$D_{mn}\left(b,\omega \right)=\sqrt{\frac{n!}{m!}}\;e^{-{\left|z\right|}^{2}/2}\;z^{m-n}\;L_{n}^{\left(m-n\right)}{\left(|z\right|}^{2})\;,\;for\;m\geq n\;,$$

with $L_{n}^{\left(m-n\right)}\left(u\right)=\frac{m!}{n!}{\left(-u\right)}^{n-m}L_{m}^{\left(n-m\right)}\left(u\right)$ for n≥m, and in which $z:=\frac{1}{\sqrt{2}}\left(b+i\omega \right)$. Therefore, if the function Π is isotropic, i.e., $\Pi \left(b,\omega \right):=w(|z{|}^{2})$, then $Q_{0}$ is the unit-trace diagonal operator:

(85) $$Q_{0}=\sum_{n=0}^{\infty }L_{n}\left(w\right)|n\rangle \langle n|\;,$$

where

(86) $$L_{n}\left(w\right)={\left(Q_{0}\right)}_{nn}=\int_{0}^{+\infty }e^{-u/2}\;L_{n}\left(u\right)\;w\left(u\right)\;du$$

is a particular Laguerre transform [27,28] with

Ln(u)≡Ln(0)(u)=∑m=0n(−1)mnmumm!.

Since the Laguerre polynomials form an orthonormal basis of the Hilbert space $L^{2}\left(R^{+},e^{-u}du\right)$, the transform (86) is defined for all *n* and for all w(u) such that $e^{u/2}w\left(u\right)$ belongs to this space. Furthermore, if *w* is such that $L_{n}\left(w\right)\geq 0$ for all *n*, then $Q_{0}$ is a density operator. For a hint in the search of solutions to this interesting problem, *w* can be viewed as the Laplace transform of a nonnegative function or distribution *ℓ* with support in [1/2,∞):

(87) $$w\left(u\right)=\int_{\frac{1}{2}}^{+\infty }e^{-ut}\;\ell \left(t\right)\;dt\;.$$

Next, from the integral formula [26],

(88) $$\begin{array}{cc} \int_{0}^{\infty }\;e^{-\nu x}\;x^{\lambda }\;L_{n}^{\alpha }\left(x\right)dx & =\frac{\Gamma (\lambda +1)\Gamma (\alpha +n+1)}{n!\;\Gamma (\alpha +1)}\nu^{-\lambda -1}\\ \; & \times {}_{2}F_{1}\left(-n,\lambda +1;\alpha +1;\nu^{-1}\right)\;, \end{array}$$

and assuming that the Frobenius theorem is valid here, we derive

(89) $$L_{n}\left(w\right)=\frac{1}{2}\int_{0}^{+\infty }e^{-(n+1/2)\Theta }\;\ell \left(t\left(\Theta \right)\right)\;\frac{d\Theta }{\Theta^{2}}\;,\;t\left(\Theta \right)=\frac{1}{2}\coth\left(\frac{1}{2\Theta }\right)\;.$$

This results in the convex integral of the Boltzmann–Planck-like density operators ρ(Θ) defined in (81):

(90) $$Q_{0}=\frac{1}{2}\int_{0}^{+\infty }\rho \left(\Theta \right)\;e^{-\frac{1}{2\Theta }}\ell \left(t\left(\Theta \right)\right)\;\frac{d\Theta }{\Theta^{2}}\;.$$

## 6. The Eight-Dimensional Case and General Relativity

Hereafter, we restrict our quantization procedure to those based on the choice of a probe ψ. We remind the reader that the Gabor quantization of functions or distributions f(x,k) on the phase space $\left\{\left(x,k\right)\in R^{8}\right\}$ is based on the overcompleteness (5) of the family of translated and modulated unit-norm probes $\psi_{x,k}\left(y\right)=e^{ik\cdot y}\psi \left(y-x\right)$:

(91) $$f\left(x,k\right)\mapsto A_{f}=\frac{1}{{\left(2\pi \right)}^{4}}\int_{R^{8}}d^{4}x\;d^{4}k\;f\left(x,k\right)\;|\psi_{x,k}\rangle \langle \psi_{x,k}|\;.$$

$A_{f}$ acts in the Hilbert space of finite-energy signals $s\left(x\right)\in H=L^{2}\left(R^{4},d^{4}x\right)$ as the integral operator

(92) $$s\left(x\right)\mapsto \left(A_{f}s\right)\left(x\right)=\langle \delta_{x}|A_{f}|s\rangle =\int_{R^{4}}d^{4}x^{\prime }\;A_{f}\left(x,x^{\prime }\right)\;s\left(x^{\prime }\right)\;,$$

with the integral kernel given by

(93) $$A_{f}\left(x,x^{\prime }\right)=\frac{1}{4\pi^{2}}\int_{R^{4}}d^{4}y\;{\hat{f}}_{k}\left(y,x^{\prime }-x\right)\;\psi \left(x-y\right)\;\bar{\psi (x^{\prime }-y)}\;.$$

Here, ${\hat{f}}_{k}\left(x,y\right)$ is the partial Fourier transform with respect to the four-vector variable *k*:

(94) $${\hat{f}}_{k}\left(x,y\right)=\frac{1}{4\pi^{2}}\int_{R^{4}}d^{4}k\;f\left(x,k\right)\;e^{-ik\cdot y}\;.$$

Remember the non-conventional sign in the Fourier exponential due to our choice of space-time metric. The Gabor semi-classical phase-space portrait of $A_{f}$ is given by

(95) $$\overset{ˇ}{f}\left(x,k\right)=\int_{R^{8}}\frac{d^{4}x^{\prime }\;d^{4}k^{\prime }}{{\left(2\pi \right)}^{4}}\;f\left(x^{\prime },k^{\prime }\right)\;{\left|\langle \psi_{x,k}|\psi_{x^{\prime },k^{\prime }}\rangle \right|}^{2}\;.$$

Explicit forms of $\overset{ˇ}{f}$ are analogous to the time-frequency case (43) apart from the fact that their writing might become quite cumbersome. Here, we are essentially concerned with the quantization and semi-classical portraits of fields on space-time, like the metric field of GR. Therefore, the Gabor quantization of the field u(x) yields the multiplication operator

(96) $$\left(A_{u}s\right)\left(x\right)=\left(u*{\left|\psi \right|}^{2}\right)\left(x\right)\;s\left(x\right)\;,$$

and its semi-classical portrait is given by

(97) $$\overset{ˇ}{u}\left(x\right)=\left(u*\left({\left|\psi \right|}^{2}*{\left|\tilde{\psi }\right|}^{2}\right)\right)\left(x\right)=\left(u*R_{{\left|\psi \right|}^{2}{\left|\psi \right|}^{2}}\right)\left(x\right)\;.$$

Here, convolution and autocorrelations are for functions on $R^{4}$ equipped with the Lebesgue measure $d^{4}x$:

(98) $$\begin{array}{cc} \left(f*g)(x\right) & =\int_{R^{4}}d^{4}y\;f\left(x-y\right)\;g\left(y\right)\;, \end{array}$$

(99) $$\begin{array}{cc} R_{\psi \psi }\left(x\right) & =\int_{R^{4}}d^{4}x^{\prime }\;\psi \left(x^{\prime }\right)\;\bar{\psi (x^{\prime }-x)}=\left(\psi *\bar{\tilde{\psi }}\right)\left(x\right)\;, \end{array}$$

where, as a reminder, $\tilde{\psi }\left(x\right)=\psi \left(-x\right)$. Repeating (31) and (45), the space-time variables acquire the status of quantum observables acting on the space of signals:

(100) $$\begin{array}{cc} x^{\mu }\mapsto A_{x^{\mu }} & =X^{\mu }-{\langle X^{\mu }\rangle }_{\psi }\;𝟙\;,\;X^{\mu }\left(s\right)\left(x\right)=x^{\mu }s\left(x\right)\;, \end{array}$$

(101) $$\begin{array}{cc} \overset{ˇ}{x^{\mu }} & =x^{\mu }-{\langle x^{\mu }\rangle }_{R_{{\left|\psi \right|}^{2}{\left|\psi \right|}^{2}}}\;. \end{array}$$

With the choice of the Gaussian probe

(102) $$G_{\sigma }\left(x\right)=\prod_{\mu =0,1,2,3}G_{\sigma_{\mu }}\left(x^{\mu }\right)\;,$$

the above formula assumes simple expressions:

(103) $$\begin{array}{cc} \left(A_{u}s\right)\left(x\right) & =\left(u*G_{\sigma }^{2}\right)\left(x\right)\;s\left(x\right)\;,\;\sqrt{\sigma }:=\prod_{\mu =0,1,2,3}\sqrt{\sigma_{\mu }}\;, \end{array}$$

(104) $$\begin{array}{cc} \overset{ˇ}{u}\left(x\right) & =\left(u*\left(G_{\sigma }^{2}*G_{\sigma }^{2}\right)\right)\left(x\right)=\left(u*G_{\sqrt{2}\sigma }^{2}\right)\left(x\right)\;. \end{array}$$

The above formalism allows us to provide a quantum version of the metric fields $\left(g_{\mu \nu }\left(x\right)\right)$ of general relativity,

(105) $$g_{\mu \nu }\left(x\right)\mapsto A_{g_{\mu \nu }}\;,$$

and to provide a regularized version depending of the choice of probe,

(106) $${\overset{ˇ}{g}}_{\mu \nu }\left(x\right)\;.$$

As a first example, let us examine the Minkowskian metric in Cartesian coordinates, $\left(g_{\mu \nu }\right)=\mathrm{diag}\left(1,-1,-1,-1\right)$. We trivially obtain $\left(A_{g_{\mu \nu }}\right)=\mathrm{diag}\left(𝟙,-𝟙,-𝟙,-𝟙\right)$. Let us now examine what happens if one quantizes the same metric as is expressed in cylindric coordinates $(g_{\mu \nu }^{\mathrm{cyl}}\left(x^{0},\rho ,\theta ,x^{3}\right)=\mathrm{diag}\left(1,-1,-\rho^{2},-1\right)$. This metric is singular in the sense that its determinant cancels on the third axis ρ=0. Due to the rotational symmetry about this axis, we naturally choose $\sigma =(\sigma_{0},\sigma ,\sigma ,\sigma_{3})$. We then find the new metric operator and its semi-classical portrait:

(107) $$\begin{array}{cc} \left(A_{g_{\mu \nu }}\right) & =\mathrm{diag}\left(𝟙,-𝟙,-\left({\hat{\rho }}^{2}+\sigma^{2}𝟙\right),-𝟙\right)\;,\;{\hat{\rho }}^{2}={\left(X^{1}\right)}^{2}+{\left(X^{2}\right)}^{2}\;,\\ ds^{2} & =d{x_{0}}^{2}-d\rho^{2}-\left(\rho^{2}+2\sigma^{2}\right)d\theta^{2}-d{x_{3}}^{2}\;. \end{array}$$

Both expressions have become regular on the third axis.

In the case of isotropic space, spherical coordinates for the spatial part are privileged, i.e., $(g_{\mu \nu }^{\mathrm{spher}}\left(x^{0},r,\theta ,\phi \right)=\mathrm{diag}\left(1,-1,-r^{2},-r^{2}\sin^{2}\theta \right)$. This metric is singular as its determinant cancels at the origin r=0. Choosing $\sigma =(\sigma_{0},\sigma ,\sigma ,\sigma )$, for the metric operator and its semi-classical portrait, we obtain

(108) $$\begin{array}{cc} \left(A_{g_{\mu \nu }}\right) & =\mathrm{diag}\left(𝟙,-𝟙,-\left({\hat{r}}^{2}+\frac{3}{2}\sigma^{2}𝟙\right),-\left({\hat{\rho }}^{2}+\sigma^{2}\right)𝟙\right)\;,\\ ds^{2} & =d{x_{0}}^{2}-dr^{2}-\left(r^{2}+3\sigma^{2}\right)d\theta^{2}-\left(\rho^{2}+2\sigma^{2}\right)d\phi^{2}\;, \end{array}$$

with ${\hat{\rho }}^{2}={\left(X^{1}\right)}^{2}+{\left(X^{2}\right)}^{2}$. Both expressions are now regular at the origin.

The reader should be aware that simply adding a positive constant to $\rho^{2}$ (cylindrical case) and to $\rho^{2}$ and $r^{2}$ (spherical case) creates non-trivial changes to the Euclidean geometry of the spatial part of the metric. Deformations of geodesics from straight lines involve elliptic functions [29].

Next, if the metric fields are known solutions of the Einstein equations for a given tensor energy density

(109) $$R_{\mu \nu }-\frac{1}{2}R=\kappa \;T_{\mu \nu }\;,\;\kappa =\frac{8\pi G}{c^{4}}\;,$$

then their respective ψ regularized versions give rise to the modified tensor energy density ${\overset{ˇ}{T}}_{\mu \nu }$ through the equation

(110) $${\overset{ˇ}{T}}_{\mu \nu }=\frac{1}{\kappa }\left({\overset{ˇ}{R}}_{\mu \nu }-\frac{1}{2}\overset{ˇ}{R}\right)\;.$$

This procedure offers the opportunity to work with smooth versions of the metric field, where the smoothing has a probabilistic content. Moreover, the notion of empty space becomes, mathematically, completely idealistic, since any quantization of the above Gabor type introduces (fictitious or real) matter, however minute it may be. This matter arises from the lack of information encoded in the parameters of the probe ψ.

The important point to be examined is the physical meaning of the set of parameters κ introduced in Section 2, or in more illustrative terms, the set σ of Gaussian widths introduced in (102). The probabilistic nature of these parameters should reflect our inability to reach exactness in terms of the information about the observed system through the data and interpretative model. There are limit values assumed by these parameters beyond which the mathematical model loses its physical relevance, i.e., where no measurement or observation makes sense. Those limit values should be viewed as based on or originating from the existence of a unique quantum of information, existing in addition to other elementary quanta, as listed by one of our group in [30,31]. It would be fruitful to revisit the present approach from a more informational viewpoint. See, for instance, ref. [32] for a survey of the employed concepts and [33,34,35,36] for a more specific viewpoint à la Souriau based on the symmetry of considered manifolds, the simplest one being the phase space $R^{8}$. We have already tackled the subject with the expressions (81) and (90).

## 7. The Example of the Uniformly Accelerated Reference System

In their illuminating 1935 article [17], Einstein and Rosen state:

*“Every field, in our opinion, must therefore adhere to the fundamental principle that singularities of the field are to be excluded.”*

It is needless to say that we agree with this statement, and the first example they provide gives us the opportunity to show how Gabor quantization of the metric field solves the singularity problem. Following the Principle of Equivalence, one can view a uniformly accelerated reference system as at rest, provided that there is a homogeneous gravitational field in space with respect to it. The corresponding metric field is given by

(111) $$ds^{2}=\alpha^{2}x_{1}^{2}dx_{0}^{2}-dx_{1}^{2}-dx_{2}^{2}-dx_{3}^{2}\;.$$

The singularity holds on the hyperplane $x_{1}=0$. For $x_{1}\neq 0$, this leads to the Einstein field equations

(112) $$R_{\mu \nu }=0\;.$$

The restriction $x_{1}\neq 0$ is necessary since the Ricci tensor is indeterminate on the hyperplane $x_{1}=0$. In view of regularization, the authors of [17] proposed to modify the metric (111) with the introduction of a small constant ς as

(113) $$ds^{2}=\left(\alpha^{2}x_{1}^{2}+\varsigma \right)dx_{0}^{2}-dx_{1}^{2}-dx_{2}^{2}-dx_{3}^{2}\;.$$

This ansatz gives rise to a regular metric and a new stress-energy tensor ${\overset{ˇ}{T}}_{\mu \nu }$ through the gravitational equation with source

(114) $${\overset{ˇ}{R}}_{\mu \nu }-\frac{1}{2}g_{\mu \nu }\;\overset{ˇ}{R}=\kappa \;{\overset{ˇ}{T}}_{\mu \nu }\;,\;\kappa =\frac{8\pi G}{c^{4}}\;,$$

whose nonvanishing components read

(115) $${\overset{ˇ}{T}}_{22}={\overset{ˇ}{T}}_{33}=-\frac{\alpha^{2}\varsigma }{\kappa }{\left(\alpha^{2}x_{1}^{2}+\varsigma \right)}^{-2}\;,$$

which cancels at ς=0, as expected. This fictitious or real stress-energy tensor is pure pressure in Directions 2 and 3.

The Einstein-Rosen ansatz is not an ansatz any longer if we apply the Gabor quantization procedure to the metric field in (111) and the resulting semi-classical portraits. This is why we checked the quantities appearing in (114). To simplify, we choose the Gaussian probe (102). From (40), we obtain for the metric operators

(116) $$\left(A_{g_{\mu \nu }}\right)=\mathrm{diag}\left(\alpha^{2}X_{1}^{2}+\alpha^{2}\frac{\sigma_{1}^{2}}{2}𝟙,-𝟙,-𝟙,-𝟙\right)\;,\;X_{1}s\left(x\right)=x_{1}s\left(x\right)\;,$$

and for their semi-classical portrait,

(117) $$\left({\overset{ˇ}{g}}_{\mu \nu }\right)=\mathrm{diag}\left(\alpha^{2}x_{1}^{2}+\alpha^{2}\sigma_{1}^{2},-1,-1,-1\right)\;.$$

Thus, the Einstein–Rosen parameter can be identified as

(118) $$\varsigma =\alpha^{2}\sigma_{1}^{2}$$

and is interpreted as proportional to the Gaussian variance for the variable $x_{1}$. Hence, (115) becomes

(119) $${\overset{ˇ}{T}}_{22}={\overset{ˇ}{T}}_{33}=-\frac{\sigma_{1}^{2}}{\kappa }{\left(x_{1}^{2}+\sigma_{1}^{2}\right)}^{-2}\;.$$

The regularization parameter $\sigma_{1}$ conveys our degree of ignorance of what the “exact” geometric modeling of the above homogeneous gravitational field in space is.

## 8. The Example of the Schwarzschild Metric Field

We now turn our attention to the Schwarzschild solution for the static spherically symmetric field produced by a spherical symmetric body at rest:

(120) $$\begin{array}{cc} ds^{2} & =\left(1-\frac{2m}{r}\right)dt^{2}-{\left(1-\frac{2m}{r}\right)}^{-1}dr^{2}-r^{2}d\theta^{2}-r^{2}\sin^{2}\theta d\phi^{2}\\ & \equiv Udt^{2}-Vdr^{2}-r^{2}d\theta^{2}-r^{2}\sin^{2}\theta d\phi^{2}\;, \end{array}$$

with appropriate units. Note that the metric for the de Sitter space-time with the horizon has the same form if 2m/r is replaced with $\Lambda r^{2}/3$ in $g_{00}$ and $g_{rr}$, where Λ is the positive cosmological constant. The cosmological horizon holds at $r=\sqrt{3/\Lambda }$.

Since the Schwarzschild solution holds outside the surface of the body that is producing the field, i.e., for 2m<r<∞, and since r=0 and r=2m are singularities (pseudo for the latter since the determinant of the metric does not cancel at r=2m), we have to determine whether our approach is able to regularize these singularities to a certain extent. Let us pick a regular isotropic ψ such that ${\left|\psi \right|}^{2}\left(x\right)\equiv {\left|\psi \right|}^{2}\left(t,r\right)$ and, consequently, $R_{{\left|\psi \right|}^{2}{\left|\psi \right|}^{2}}\left(r\right)$ are probability distributions. Applying (96) and (97) yields the metric multiplication operators

(121) $$\begin{array}{cc} \left(A_{U}s\right)\left(x\right) & =\left(U*{\left|\psi \right|}^{2}\right)\left(x\right)\;s\left(x\right)=\left(1-\frac{2m}{r}+2m{\langle Y_{r}\left(r^{\prime }\right)\left(\frac{1}{r}-\frac{1}{r^{\prime }}\right)\rangle }_{{\left|\psi \right|}^{2}}\right)s\left(x\right)\\ & \equiv {\tilde{U}}_{{\left|\psi \right|}^{2}}\left(r\right)\;s\left(x\right) \end{array}$$

(122) $$\begin{array}{cc} \left(A_{V}s\right)\left(x\right) & =\left(V*{\left|\psi \right|}^{2}\right)\left(x\right)\;s\left(x\right)=\left(1+\frac{2m}{r}+\frac{2m}{r}{\langle \frac{m}{r^{\prime }}\ln\frac{|r+r^{\prime }-2m|}{|r-r^{\prime }-2m|}\rangle }_{{\left|\psi \right|}^{2}}\right.\\ & \left.+2m{\langle Y_{r}\left(r^{\prime }\right)\left(\frac{1}{r^{\prime }}-\frac{1}{r}+\frac{m}{rr^{\prime }}\ln\frac{|r-r^{\prime }-2m|}{|r^{\prime }-r-2m|}\right)\rangle }_{{\left|\psi \right|}^{2}}\right)s\left(x\right)\\ & \equiv {\tilde{V}}_{{\left|\psi \right|}^{2}}\left(r\right)\;s\left(x\right)\equiv \left(2-{\tilde{U}}_{{\left|\psi \right|}^{2}}\left(r\right)+L_{{\left|\psi \right|}^{2}}\left(r\right)\right)\;s\left(x\right) \end{array}$$

(123) $$\begin{array}{cc} \left(A_{r^{2}}s\right)\left(x\right) & =\left(r^{2}*{\left|\psi \right|}^{2}\right)\left(x\right)\;s\left(x\right)=\left(r^{2}+{\langle {r^{\prime }}^{2}\;\rangle }_{{\left|\psi \right|}^{2}}\right)s\left(x\right)\;, \end{array}$$

(124) $$\begin{array}{cc} \left(A_{r^{2}\sin^{2}\theta }s\right)\left(x\right) & =\left(r^{2}\sin^{2}\theta *{\left|\psi \right|}^{2}\right)\left(x\right)\;s\left(x\right)=\left(r^{2}\sin^{2}\theta +\frac{2}{3}{\langle {r^{\prime }}^{2}\;\rangle }_{{\left|\psi \right|}^{2}}\right)s\left(x\right)\;, \end{array}$$

after regularization of logarithmic singularity through the Cauchy principal value in (122). We have primed the integration variable in the computation of expected values in order to avoid the confusion with the external variable r=∥x∥, and we have introduced the Heaviside function $Y_{r}\left(r^{\prime }\right):=Y\left(r^{\prime }-r\right)$. Note that the 2/3 in (124) is the average of $\sin^{2}\theta$ on the sphere. We remind the reader that the notation ${\langle f\rangle }_{p}$ stands for the expected value of the function *f* with respect to the probability distribution *p*.

The corresponding semi-classical portraits have similar expressions provided that we replace ${\left|\psi \right|}^{2}$ with $R_{{\left|\psi \right|}^{2}{\left|\psi \right|}^{2}}$:

(125) $$\begin{array}{cc} \overset{ˇ}{U}\left(x\right) & =\left(U*R_{{\left|\psi \right|}^{2}{\left|\psi \right|}^{2}}\right)\left(x\right)={\tilde{U}}_{R_{{\left|\psi \right|}^{2}{\left|\psi \right|}^{2}}}\left(r\right)\;, \end{array}$$

(126) $$\begin{array}{cc} \overset{ˇ}{V}\left(x\right) & =\left(V*R_{{\left|\psi \right|}^{2}{\left|\psi \right|}^{2}}\right)\left(x\right)={\tilde{V}}_{R_{{\left|\psi \right|}^{2}{\left|\psi \right|}^{2}}}\left(r\right)\;, \end{array}$$

(127) $$\begin{array}{cc} \overset{ˇ}{r^{2}}\left(x\right) & =\left(r^{2}*R_{{\left|\psi \right|}^{2}{\left|\psi \right|}^{2}}\right)\left(x\right)=r^{2}+{\langle {r^{\prime }}^{2}\;\rangle }_{R_{{\left|\psi \right|}^{2}{\left|\psi \right|}^{2}}}\;, \end{array}$$

(128) $$\begin{array}{cc} \overset{ˇ}{r^{2}\sin^{2}\theta }\left(x\right) & =\left(r^{2}\sin^{2}\theta *R_{{\left|\psi \right|}^{2}{\left|\psi \right|}^{2}}\right)\left(x\right)=r^{2}\sin^{2}\theta +\frac{2}{3}{\langle {r^{\prime }}^{2}\;\rangle }_{R_{{\left|\psi \right|}^{2}{\left|\psi \right|}^{2}}}\;. \end{array}$$

Here, we analyze the functions ${\tilde{U}}_{p}\left(r\right)$ and ${\tilde{V}}_{p}\left(r\right)$, where p=p(t,r) denotes a spatially isotropic probability distribution on $R^{4}$, as are ${\left|\psi \right|}^{2}$ or $R_{{\left|\psi \right|}^{2}{\left|\psi \right|}^{2}}$. We write their expressions after rearranging some terms:

(129) $$\begin{array}{cc} {\tilde{U}}_{p}\left(r\right) & =1-\frac{2m}{r}+2m{\langle Y_{r}\left(r^{\prime }\right)\left(\frac{1}{r}-\frac{1}{r^{\prime }}\right)\rangle }_{p}=1-\frac{2m}{r}{\langle {𝟙}_{\left[0,r\right]}\left(r^{\prime }\right)\rangle }_{p}-2m{\langle Y_{r}\left(r^{\prime }\right)\frac{1}{r^{\prime }}\rangle }_{p}\;, \end{array}$$

(130) $$\begin{array}{cc} {\tilde{V}}_{p}\left(r\right) & =2-{\tilde{U}}_{p}\left(r\right)+L_{p}\left(r\right)\;, \end{array}$$

with

(131) $$\begin{array}{cc} L_{p}\left(r\right) & =\frac{2m^{2}}{r}{\langle \frac{1}{r^{\prime }}\left[\ln\frac{|r^{\prime }+\left(r-2m\right)|}{|r^{\prime }-\left(r-2m\right)|}+Y_{r}\left(r^{\prime }\right)\ln\frac{|r^{\prime }-\left(r-2m\right)|}{|r^{\prime }-\left(r+2m\right)|}\right]\rangle }_{p} \end{array}$$

(132) $$\begin{array}{cc} \; & =\frac{2m^{2}}{r}{\langle \frac{1}{r^{\prime }}\left[{𝟙}_{\left[0,r\right]}\left(r^{\prime }\right)\ln\frac{|r^{\prime }+\left(r-2m\right)|}{|r^{\prime }-\left(r-2m\right)|}+Y_{r}\left(r^{\prime }\right)\ln\frac{|r^{\prime }+\left(r-2m\right)|}{|r^{\prime }-\left(r+2m\right)|}\right]\rangle }_{p}. \end{array}$$

Regularization at the Schwarzschild Radius Value

We note the regularization of the metric at the classical singularity r=2m:

(133) $$\begin{array}{cc} {\tilde{U}}_{p}\left(2m\right) & ={\langle Y_{2m}\left(r^{\prime }\right)\left(1-\frac{2m}{r^{\prime }}\right)\rangle }_{p}>0\;, \end{array}$$

(134) $$\begin{array}{cc} {\tilde{V}}_{p}\left(2m\right) & =1+{\langle {𝟙}_{\left[0,2m\right]}\left(r^{\prime }\right)\rangle }_{p}+{\langle Y_{2m}\left(r^{\prime }\right)\left(\frac{2m}{r^{\prime }}+\frac{m}{2r^{\prime }}\ln\frac{r^{\prime }}{|r^{\prime }-4m|}\right)\rangle }_{p}>0\;. \end{array}$$

Behaviour at Large *r*

Trivially, we have

(135) $${\tilde{U}}_{p}\left(r\right)\underset{r\to \infty }{\to }1\;,\;{\tilde{V}}_{p}\left(r\right)\underset{r\to \infty }{\to }1\;.$$

Behaviour at r=0

Less trivially, we have

(136) $${\tilde{U}}_{p}\left(r\right)\underset{r\to 0}{\to }1-{\langle \frac{2m}{r^{\prime }}\rangle }_{p}\;,\;{\tilde{V}}_{p}\left(r\right)\underset{r\to 0}{\to }1+{\langle \frac{2m}{r^{\prime }}\rangle }_{p}+4m^{2}{\langle \frac{1}{r^{\prime }\left(r^{\prime }-2m\right)}\rangle }_{p}\;,$$

where the last mean value should be understood in the sense of the Cauchy principal value, or more precisely Hilbert transform, for the integral

(137) $${\langle \frac{1}{r^{\prime }(r^{\prime }-2m}\rangle }_{p}=4\pi \int_{-\infty }^{+\infty }dt^{\prime }\int_{0}^{+\infty }dr^{\prime }\frac{r^{\prime }p\left(t^{\prime },r^{\prime }\right)}{r^{\prime }-2m}=-4\pi^{2}\int_{-\infty }^{+\infty }dt^{\prime }\;H(Y\left(\cdot \right)\left(\cdot \right)p\left(t^{\prime },\left(\cdot \right)\right)\left(2m\right)\;,$$

with the notation

(138) $$H\left(u\left(\cdot \right)\right)\left(t\right)=\frac{1}{\pi }\;p.v.\int_{-\infty }^{+\infty }\frac{u(\tau )}{t-\tau }\;d\tau \;.$$

The positiveness of the derivative of ${\tilde{U}}_{p}$,

(139) $$\frac{d{\tilde{U}}_{p}}{dr}\left(r\right)=\frac{2m}{r^{2}}\;{\langle {𝟙}_{\left[0,r\right]}\left(r^{\prime }\right)\rangle }_{p}>0\;,$$

entails that ${\tilde{U}}_{p}\left(r\right)$ is monotonically increasing from $U_{\min}=1-{\langle \frac{2m}{r^{\prime }}\rangle }_{p}$ (at which the slope is infinite) to 1. Hence, one deals with two cases.

1. If $U_{\min}>0$, i.e., ${\langle \frac{2m}{r^{\prime }}\rangle }_{p}<1$, then the temporal term of the Schwarzschild metric is completely regularized.
2. If $U_{\min}\leq 0$, i.e., ${\langle \frac{2m}{r^{\prime }}\rangle }_{p}\geq 1$, then there is a smaller “Schwarzschild radius” $r_{s0}\in \left(0,2m\right)$ for the temporal part, defined by the equation
  (140) $$r_{s0}=\frac{2m{\langle {𝟙}_{\left[0,r_{s0}\right]}\left(r^{\prime }\right)\rangle }_{p}}{1-2m{\langle Y_{r_{s0}}\left(r^{\prime }\right)\frac{1}{r^{\prime }}\rangle }_{p}}\;.$$

The situation is less obvious for the radial metric term ${\tilde{V}}_{p}\left(r\right)$. Nevertheless, through a suitable choice of probability distribution, one can expect a full regularization, too.

A more developed analysis with explicit examples together with the determination of the geodesics and of the stress-energy tensor ${\overset{ˇ}{T}}_{\mu \nu }$ resulting in this regularization and its interpretation will be addressed in a forthcoming work.

## 9. Final Discussion

Let us initiate the discussion by quoting the authors of [37]:

*“… infinities in General relativity come in the form of singularities [38] and they point to the breakdown of our current understanding of gravity. The standard view in the community is that quantum gravity should be able to resolve this issue by smoothing out singularities. Nonetheless, despite the many existing approaches to quantum gravity, there is no consensus about what it is and how one should construct a quantum theory of spacetime, thus a proof of principle for the singularity avoidance is yet to be found.”*

The present work should be viewed as a partial contribution to this program. We have presented a non-orthodox quantization that transforms functions on the eight-dimensional phase space (time-space frequency-wave vector) into operators in the Hilbert space of signals on space-time, or, equivalently, in the Hilbert space of their Fourier transforms. Non-orthodox means that there is no Planck constant involved in our approach, so there is no associated particle, no mass, no energy, and no momentum associated with the resulting quantum objects. We remind the reader that, with the Planck constant, one enters the domain of quantum mechanics and quantum field theory with its Fock space formalism. Its fundamental role is to bridge two worlds, for instance, the world of classical waves with its phase space (time-space frequency-wave vector) and the quantum mechanics built from the classical phase space (space and momentum).

As a first exploration, we have considered the metric field of general relativity, but we could as well examine the Maxwell electromagnetic field. These fields are functions of space-time coordinates, but nothing prevents us from extending their variable domain with the inclusion of frequencies and wave vectors. The latter could be viewed as phenomenological quantities, analogous to those introduced in various studies, e.g., waves in plasmas.

Concerning general relativity, we have shown that the quantization of a metric field that is a solution to the Einstein equation in empty space gives rise to a regularized version of it and to a stress-energy tensor. Although the latter could be thought as “fictitious”, we believe, on the contrary, that its existence is inescapable due to our ignorance of an “exact” mathematical model for describing gravitation. In this regard, the non-emptiness of space-time is a type of attribute that might appear to physicists as idealistic or superfluous, while it is inescapable as a completion of any mathematical model.

Finally, one could think that our approach of transforming space-time points and metric fields into operators is a parent of non-commutative geometry, as is nicely presented, for instance, in [39]. In fact, our quantization of the classical geometry of space-time yields a (formal) commutative algebra of multiplication operators, formally meaning that we make abstractions of domain considerations when dealing with products of such operators. On the other hand, the algebra becomes non-commutative if we quantize extensions of the metric field to functions depending on the frequency and wave vector.

## Data Availability

Not applicable.

## Abbreviations

The following abbreviations are used in this manuscript:

UIR	Unitary Irreducible Representation
POVM	Positive Operator-Valued Measure
CCR	Canonical Commutation Rule
GR	General Relativity
